# Transplantation of a three-lobed donor left lung: A case report

**DOI:** 10.1177/2050313X19834155

**Published:** 2019-03-01

**Authors:** Samuel Jacob, Ian A Makey, Magdy M El-Sayed Ahmed, Jorge M Mallea, David B Erasmus, Erol V Belli

**Affiliations:** 1 Department of Cardiothoracic Surgery, Mayo Clinic, Jacksonville, FL, USA; 2 Department of Surgery, Faculty of Medicine, Zagazig University, Zagazig, Egypt; 3 Division of Transplant Medicine, Mayo Clinic, Jacksonville, FL, USA

**Keywords:** Lung, organ donor management, transplantation

## Abstract

A true left middle lobe (lingular lobe) is very rare, but accessory fissures can be unexpectedly found at transplant. Pre-transplant knowledge of accessory lobes and accessory fissures aids in preparation, transplantation, postoperative assessment, and long-term care planning; however, fissures and accessory lobes can be overlooked by radiologists during routine evaluation of images. Here, we describe the first left lung with three anatomical lobes that was successfully transplanted into a 63-year-old patient with idiopathic pulmonary fibrosis. This anatomical variation did not change our surgical plan or technique, but surgeons should be aware of this possibility, especially when planning postoperative care.

## Introduction

A true left middle lobe is characterized by a lingular bronchus that originates from the main bronchus, separately from the left upper division; true left middle lobe is very rare.^1^ An accessory fissure is a cleft lined by two layers of visceral pleura, and it is the most common finding in the right lobe.^2^ When complete, the accessory fissure demarcates the accessory lobe, but accessory fissures are often overlooked on preoperative chest radiography and computed tomography (CT). The second most common accessory fissure described by anatomical studies is a left minor fissure, which was first described by Boyden.^3^ A complete left accessory fissure with a separate bronchus from the main bronchus can lead to a true left middle lobe, as in our presented case. Accessory fissures can occur on both sides, and its prevalence reported by anatomists ranges from 0.9% to 8%.^4–7^ However, a true three-lobed left lung has not been previously reported. In this report, we present the intraoperative characteristics of a rare, anatomical, left three-lobed variant found during a left lung transplant.

## Case report

**Figure 1.**
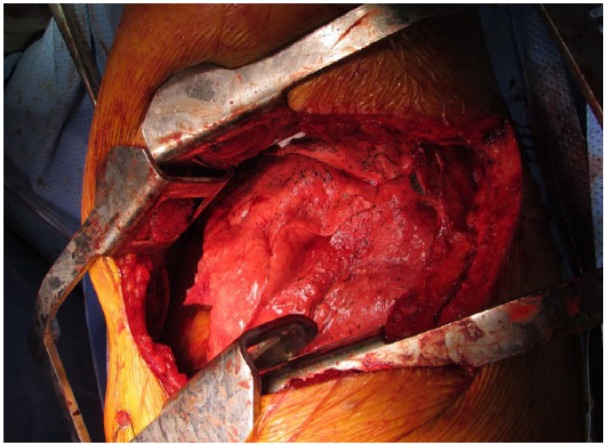
Left posterolateral thoracotomy. Intraoperative image showing the deflated left lung and its three lobes.

**Figure 2.**
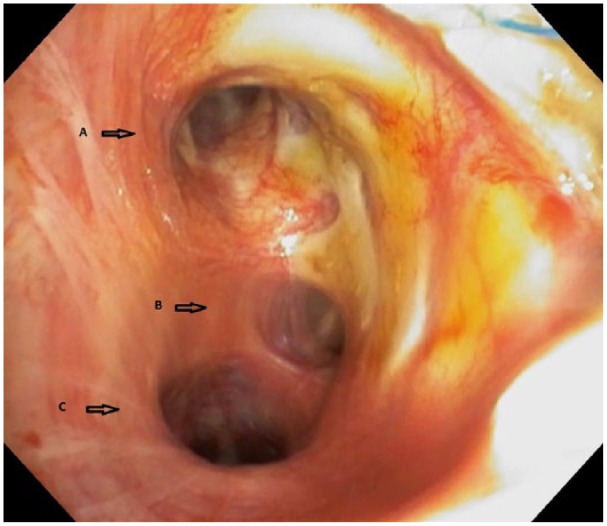
Postoperative bronchoscopic image showing the three-lobed bronchus of left lung: A, upper bronchus; B, middle accessory lob bronchus; and C, lower lob bronchus.

**Figure 3.**
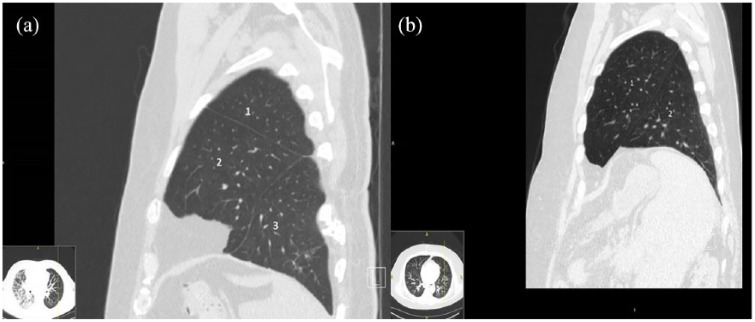
(a) Post-transplant computed tomographic image showing the three lobes of the left lung: 1, upper lob; 2, middle accessory lob bronchus; and 3, lower lob. (b) Normal variant of left lung: 1, upper lob and 2, lower lob.

A 63-year-old man with idiopathic pulmonary fibrosis was admitted to our hospital to undergo left lung transplant. Recipient anatomy was normal; donor anatomy similarly showed no abnormalities on preoperative chest radiographs and CT images; and bronchoscopic findings at procurement were reported normal. We confirmed the decision to proceed with lung transplant. The patient was intubated, placed in the right lateral decubitus position, and prepared for left lung transplant. Upon receiving the donor lung, we immediately recognized the three lobes of the left lung (Figure 1). Our immediate concern was that our team had mistakenly received the wrong organ (right lung) during packing and transportation. However, the hilar anatomy and anatomical relationships between the pulmonary arteries, pulmonary vein cuff, and bronchus were consistent with the left lung. We decided to proceed, and the presence of this anatomical variation did not change our surgical plan or technique. Left single-lung transplant was performed without cardiopulmonary bypass, and the procedure was successful and well tolerated. Post-transplant bronchoscopy showed a three-lobed bronchus (Figure 2), and CT showed three demarcated lobes (Figure 3). The patient recovered without complications, and his postoperative course was uneventful. He was discharged on postoperative day 10.

## Discussion

The left middle lobe of this lung had a complete fissure separating the lingula and upper division. Accessory fissures and pulmonary lobes are often underappreciated in donor-lung selection and misinterpreted at the time of operation.^8^ Pre-transplant knowledge of accessory lobe aids in preparation, transplantation, postoperative assessment, and long-term care planning; however, fissures and lobes can be overlooked by radiologists during routine evaluations of radiographic and CT images. The bronchopulmonary segment and arterial anatomy are often normal, which can be confirmed with bronchoscopy. In this case, the left minor fissure separated the lingula from the rest of the left upper lobe to create the left middle lobe.^8^ To our knowledge, this is the first report of a transplant of a three-lobed left lung. Surgeons should be aware of this possibility. This variant fissure of the left lung may help explain certain unusual radiographic findings observed during donor evaluation and may be a reason to reject an organ in extreme cases if radiographic findings are misinterpreted. Similarly, knowledge of this variant may help explain certain post-transplant radiographic findings, such as fluid extension into the variant fissure, and may help differentiate infection and consolidation. Moreover, awareness of this three-lobed left lung variant could help avoid confusion when the organ is received by the transplant team, who could evaluate other lung anatomical findings to differentiate left and right lungs, as well as help avoid misidentification of the left and right lungs during procurement, especially if labeling and packing lungs meant to be separated and transplanted into two recipients.

In conclusion, to our knowledge, this is the first report of a transplant of a left lung with three lobes. Presence of this anatomical variation in the donor side did not change our surgical plan or technique, but surgeons should be aware of this possibility, especially when planning postoperative care. However, finding this variant in the recipient lung might change the management and surgical planning especially if a one-lob transplant is indicated such as children group. In addition, the trimming level of the bronchus and the anastomosis might be different.
